# Supplementation of sperm cryopreservation media with H_2_S donors enhances sperm quality, reduces oxidative stress, and improves in vitro fertilization outcomes

**DOI:** 10.1038/s41598-024-62485-2

**Published:** 2024-05-30

**Authors:** Asefeh Mohammadi, Farnoosh Jafarpour, Nima Tanhaei Vash, Mehdi Hajian, Mohammad Hossein Nasr-Esfahani

**Affiliations:** 1 Department of Biology, Faculty of Science and Technology, ACECR Institute of Higher Education (Isfahan), Isfahan, Iran; 2 Department of Animal Biotechnology, Reproductive Biomedicine Research Center, Royan Institute for Biotechnology, ACECR, Isfahan, Iran

**Keywords:** Antioxidant, Cryopreservation, Goat, GYY4137, In vitro fertilization, NaHS, Sperm, Animal biotechnology, Embryology

## Abstract

Cryopreservation of sperm can cause oxidative stress and damage, leading to decreased different functional parameters and fertilization potential. In this study, we evaluated two types of H_2_S donors: NaHS, a fast-releasing donor, and GYY4137, a slow-releasing donor during cryopreservation of goat sperm. Initially, we determined that 1.5 and 3 μM NaHS, and 15 and 30 μM GYY4137 are optimal concentrations that improved different sperm functional parameters including motility, viability, membrane integrity, lipid peroxidation, and ROS production during incubation at 38.5 °C for 90 min. We subsequently evaluated the impact of the optimal concentration of NaHS and GYY4137 supplementation on various functional parameters following thawing during cryopreservation. Our data revealed that supplementation of extender improved different parameters including post-thaw sperm motility, viability, membrane integrity, and reduced DNA damage compared to the frozen-thawed control group. The supplementation also restored the redox state, decreased lipid peroxidation, and improved mitochondrial membrane potential in the thawed sperm. Finally, we found that supplementation of the extender with NaHS and GYY4137 enhanced IVF outcomes in terms of blastocyst rate and quality of blastocysts. Our results suggest that both donors can be applied for cryopreservation as antioxidants to improve sperm quality and IVF outcomes of frozen-thawed goat sperm.

## Introduction

Preservation of sperm by two common techniques including cryopreservation at − 196 °C and cold storage at 4 °C are crucial for assisted reproduction in both the farm animal industry and also in humans^1^.

Excess reactive oxygen species (ROS) produced during cryopreservation are known to have harmful effects on the structure and function of sperm^2^. Despite their harmful effects at super physiological levels a state commonly called “oxidative stress”, ROS at the physiological level, are proven to be essential for spermatogenesis including capacitation, acrosome reaction, and during sperm–oocyte fusion^3–5^. Lipid peroxidation induced by oxidative stress damages sperm membrane and leads to a decreased sperm motility and thereby hinders sperm–oocyte fusion^6^. Excess ROS, by affecting the integrity of sperm DNA, compromises the paternal genomic contribution to the newly-formed embryo^7^. It is noteworthy to mention that sperm have low antioxidant capacity due to the limited cytoplasmic volume. Additionally, high content of polyunsaturated fatty acids (PUFA) in the cell membrane makes the sperm vulnerable to oxidative condition^8^. Therefore, the regulation of redox state in the sperm is very important for maintaining its functionality during both in vivo or in vitro fertilization.

Hydrogen sulfide (H_2_S) is the third and recently discovered endogenous gasotransmitter in addition to nitric oxide (NO) and carbon monoxide (CO)^9^. The existence of endogenous H_2_S has been detected in various tissues of mammalians including brain^10,11^, spinal cord^12^, heart^13,14^, lung^15^, kidney^16,17^, liver^18^, gastrointestinal tract^19,20^ and reproductive organs^21–24^*.* This gasotransmitter is mostly biosynthesized in transsulfuration pathway. In this enzymatic pathway cystathionine beta synthase (CBS) and cystathionine γ lyase (CSE) as two pyridoxal-5′-phosphate dependent enzymes mediate production of H_2_S in the cytoplasm. In addition, 3-mercaptopyruvate sulfur transferase (3-MPST) also mediates the production of H_2_S in the mitochondria^25^.

In the male reproductive system, the expression of H_2_S synthesis enzymes has been detected in the testis^26,27^, epididymis^28^ sperm^29^, prostate^24,30^ and seminal vesicle^31^ and penile corpus cavernosum^32^. It is well established that the physiological level of H_2_S plays an important role in many physiological facets of male reproductive system including spermatogonia proliferation^26^, spermatogenesis^23^, erectile function^33–36^, blood–testis barrier^23^ and vas deferens smooth-muscle relaxation^37,38^.

In this regard, a series of studies have demonstrated that H_2_S has important physiological and cytoprotective functions in the biological systems^39,40^. Some studies have revealed some mechanisms for the regulatory effect of H_2_S on ROS production in biological systems. One of these well-known mechanisms is that H_2_S dissociate into H^+^, HS^−^ and S^2−^ and thereafter, HS^−^ quenches the free radicals and so scavenges ROS^41,42^. Besides to the scavenging role of H_2_S, this molecule can also increase intracellular glutathione (GSH) and thioredoxin (Trx-1) proteins synthesis which ultimately scavenge free radicals via nonenzymatic pathway^43,44^. Likewise, H_2_S triggers endogenous enzymatic antioxidant defense and promotes the expression of numerous enzymatic antioxidants such as superoxide dismutase (SOD), catalase (CAT), glutathione reductase (GR) and glutathione peroxidase (GPx)^40,45^. Given that H_2_S can attenuate oxidative stress and act as an antioxidant, H_2_S donors has been used in various cell lines.

H_2_S donors can be classified into two types according to their mode of action^46^: (1) fast-releasing agents: sodium hydrogen sulfide (NaHS) is one of the first donors that has been used in various biological studies^47^ and (2) slow-releasing agents: GYY4137, as a phosphorodithioate derivative, produces H_2_S in a slow and continuous release manner, which more similarly mimics the in vivo conditions^48^.

Considering the physiological and cytoprotective significance of H_2_S^49^, as well as the functional role of H_2_S donor compounds in biological systems^50^ and also in sperm^22,29,58^ to the best of our knowledge, in this study for the first time, we investigate the potential protective capabilities of fast-releasing (NaHS) and slow-releasing (GYY4137) H_2_S donors in maintenance of sperm functional parameters and in vitro fertilization rate in goat species following cryopreservation.

## Results

### Determining the optimal concentration of NaHS on sperm motility and viability

The fast-releasing donor of H_2_S, NaHS, and slow releasing donor, GYY4137, were used to determine the optimum concentration(s) for incubation at 38.5 °C at various time points based on the sperm motility and viability assessments.

**Figure 1:**
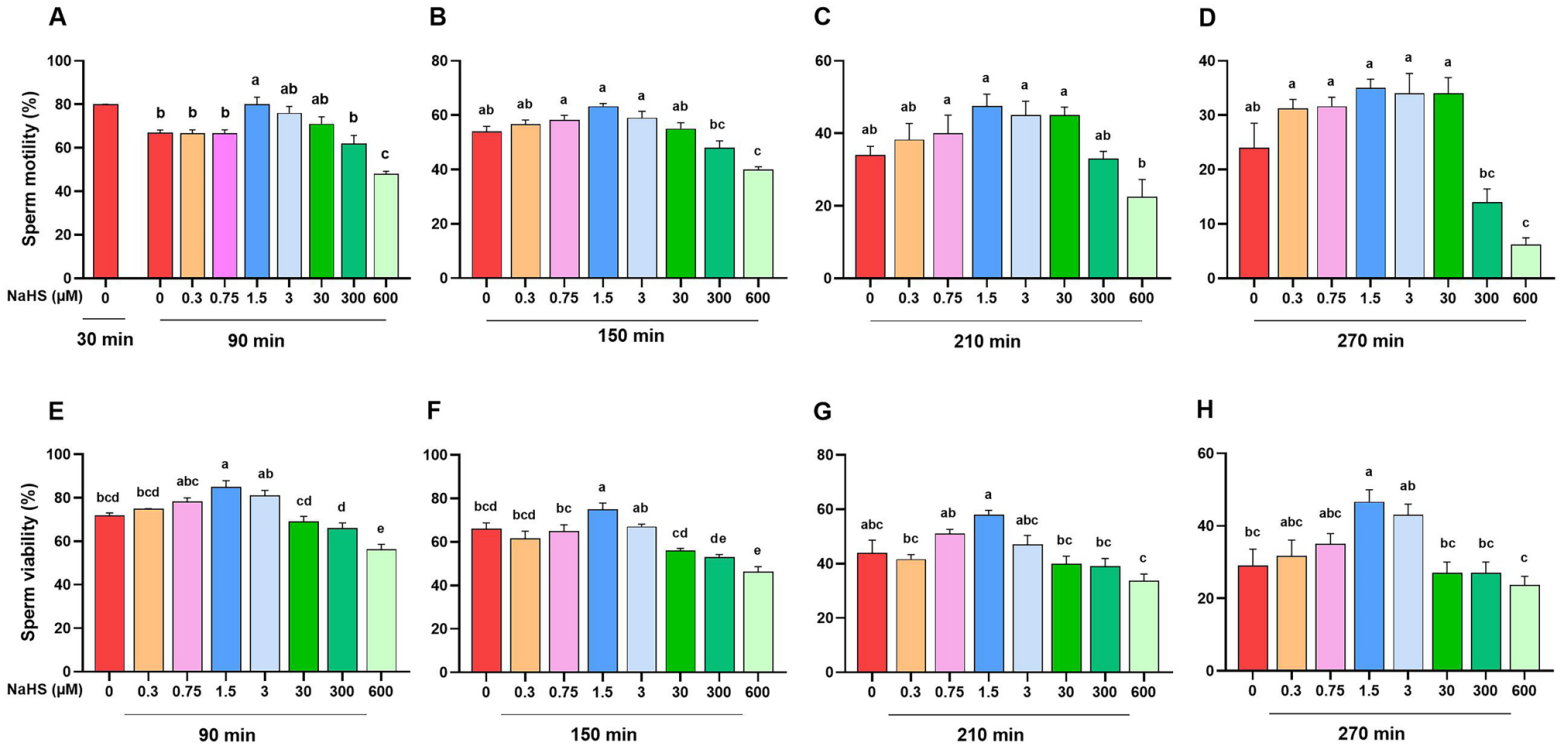
Determining the optimal concentrations of NaHS by assessing sperm motility and viability. The effect of various concentrations of NaHS (0–600 µM) on (**A**–**D**) sperm motility and (**E**–**H**) sperm viability during incubation at 38.5 °C at different time points (90, 150, 210 and 270 min) in goat species. Each column represents the mean ± standard error of mean (SEM), and different letters indicate significant differences (*P* < 0.05).

As it is depicted in Fig. 1A, the sperm motility of 1.5 µM NaHS (80.0 ± 3.4) was significantly higher than all concentrations of NaHS at 90 min except to 3 and 30 NaHS (*P* < 0.05). At 150 min time point, the sperm motility of 1.5 and 3 µM NaHS (63.3 ± 1.8 and 59.0 ± 2.5, respectively) were significantly higher than 300 and 600 µM NaHS (48.0 ± 2.6 and 40.0 ± 2.8, respectively) (*P* < 0.05, Fig. 1B). The sperm motility of 600 µM NaHS (22.5 ± 4.8) was significantly lower than 0.75, 1.5, 3 and 30 µM NaHS at 210 min (*P* < 0.05, Fig. 1C). Finally, at 270 min time point the sperm motility of 600 µM NaHS (6.2 ± 1.3) was significantly lower than all other concentrations of NaHS except for 300 µM (14.0 ± 2.5) (*P* < 0.05, Fig. 1D).

Subsequently, we also assessed the sperm viability to determine the optimum concentration of NaHS. As it is depicted in Fig. 1E, the sperm viability of 1.5 µM NaHS (85.0 ± 2.9) was significantly higher than other concentration of NaHS except for 0.75 and 3 µM NaHS at 90 min time point (*P* < 0.05). At 150 min time point, the sperm viability of 1.5 µM NaHS (75.0 ± 2.9) was significantly higher than other concentrations of NaHS except for 3 µM NaHS (67.0 ± 1.3) (*P* < 0.05, Fig. 1F). Finally, the sperm viability of 1.5 µM NaHS was significantly higher than other concentrations of NaHS except for 0.75 and 3 µM NaHS at 210- and 270-min time points (*P* < 0.05, Fig. 1G,H).

Altogether, the results of this section demonstrated that 1.5 and 3 µM concentrations of NaHS had higher motility and viability compared to other concentrations of NaHS at various time points. So, we used 1.5 and 3 µM concentrations for further assessments in this study.

### Determining the optimal concentration of GYY4137 on sperm motility and viability

**Figure 2:**
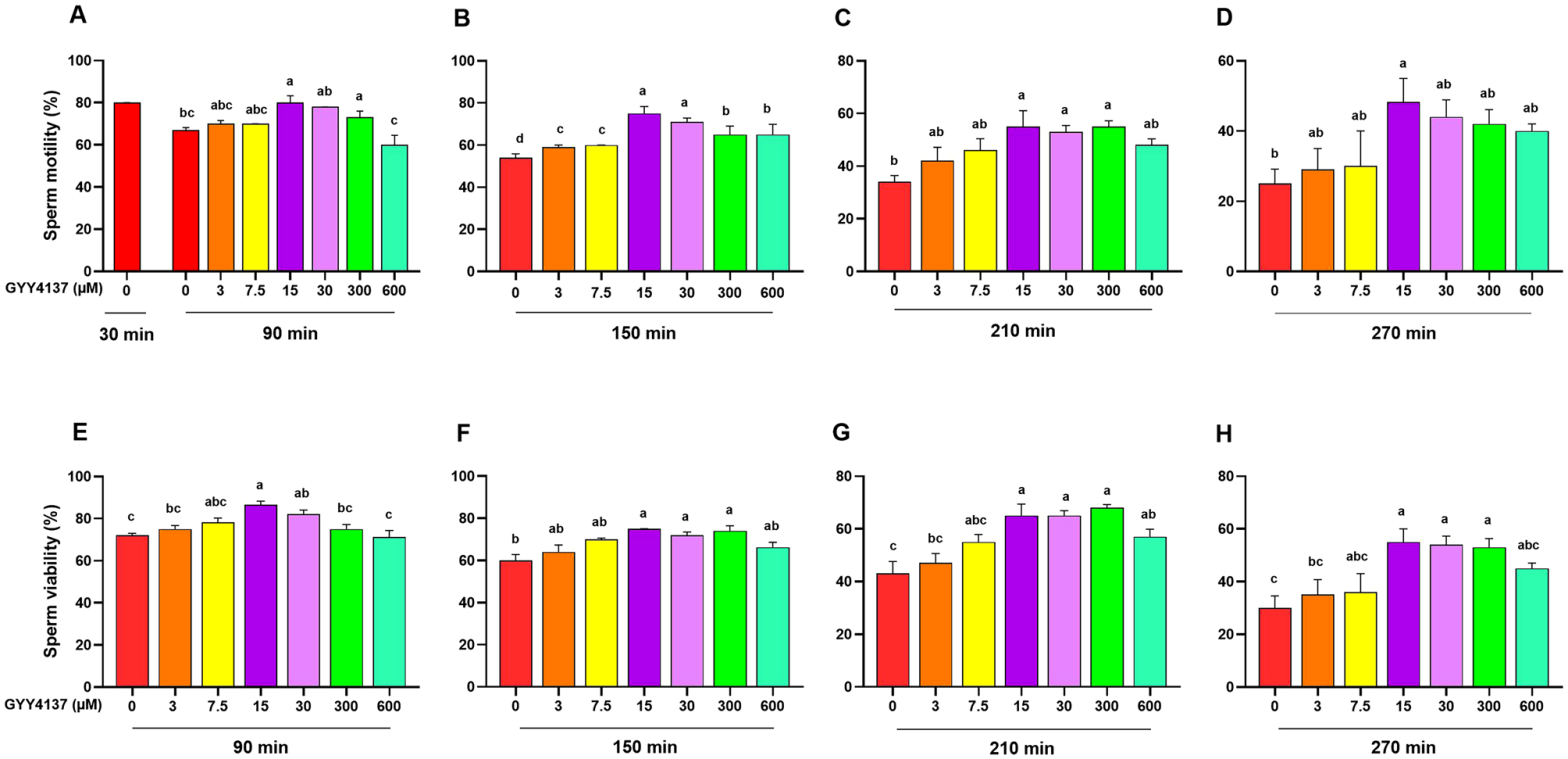
Determining the optimal concentrations of GYY4137 by assessing sperm motility and viability. The effect of various concentrations of GYY4137 (0–600 µM) on (**A**–**D**) sperm motility and (**E**–**H**) sperm viability during incubation at 38.5 °C at different time points (90, 150, 210 and 270 min) in goat species. Each column represents the mean ± standard error of mean (SEM), and different letters indicate significant differences (*P* < 0.05).

As it is revealed in Fig. 2A, the sperm motility of 15 µM GYY4137 (80.0 ± 3.4) was significantly higher than 0 and 600 µM GYY4137 at 90 min time point (*P* < 0.05). At 150 min time point, the sperm motility of 15 and 30 µM GYY4137 (75 ± 3.4 and 71 ± 1.9, respectively) was significantly higher than all other concentrations of GYY4137 (*P* < 0.05, Fig. 2B). The sperm motility of 15, 30 and 300 µM GYY4137 (55.0 ± 6, 55 ± 2.5 and 55.0 ± 2.3, respectively) was significantly higher than control at 210 min time point (*P* < 0.05, Fig. 2C). Finally, at 270 min time point the sperm motility of 15 µM GYY4137 (48.3 ± 6.7) was significantly higher than untreated group (25.0 ± 4.2) (*P* < 0.05, Fig. 2D).

Furthermore, the sperm viability was also assessed to determine the optimum concentration(s) of GYY4137. As it is shown in Fig. 2E, the sperm viability of 15 µM GYY4137 (86.7 ± 1.7) was significantly higher than 0 and 600 µM GYY4137 at 90 min time point (*P* < 0.05). At 150 min time point, the sperm viability of untreated group (60.0 ± 2.8) was significantly lower than 15, 30 and 300 µM GYY4137 (*P* < 0.05, Fig. 2F). Finally, the sperm viability of 15, 30 and 300 µM GYY4137 was significantly higher than 0 and 3 µM GYY4137 at 210- and 270-min time point (*P* < 0.05, Fig. 2G,H).

Overall, the results of this section demonstrated that 15 and 30 µM concentrations of GYY4137 had higher motility and viability compared to other concentrations of GYY4137 at various time points. So, we used these two concentrations for further assessments in this study.

Given that 1.5 and 3 μM NaHS (Fig. 1), and 15 and 30 μM GYY4137 (Fig. 2) remarkably improved the sperm motility and viability compared to the control, we assessed other functional sperm parameters following the treatment with optimal concentration of NaHS and GYY4137 at 90 min time point.

### Supplementation of extender with optimal concentrations of NaHS and GYY4137 improved the sperm membrane integrity, lipid peroxidation and ROS level during 1.5 h incubation at 38.5 °C

After determining the optimal concentrations of NaHS and GYY4137 by assessing the sperm motility and viability, we assessed the effect of optimal concentrations on other sperm functional parameters including sperm membrane integrity, lipid peroxidation and ROS level production in 38.5 °C at 90 min time point.

**Figure 3:**
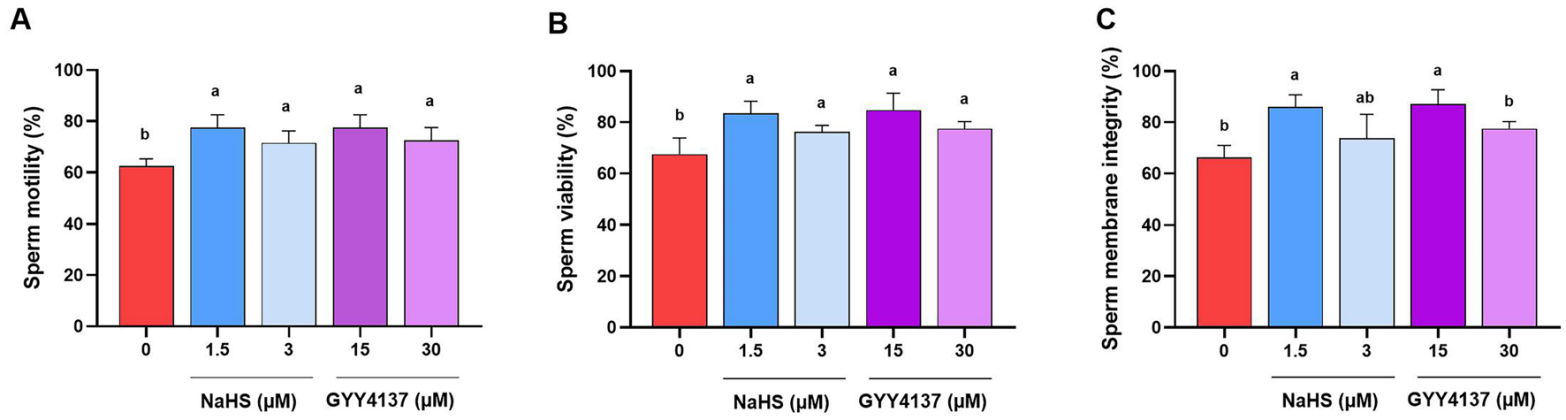
Optimal concentrations of NaHS and GYY4137 enhance functional parameters during in vitro incubation. The effect of optimal concentrations of NaHS (1.5 and 3 µM) and GYY4137 (15 and 30 µM) on (**A**) sperm motility, (**B**) viability and (**C**) membrane integrity during 1.5 h incubation at 38.5 °C in goat species. Each column represents the mean ± standard error of mean (SEM), and different letters indicate significant differences (*P* < 0.05).

As it is revealed in Fig. 3A,B, in a new experiment, we assessed the optimal concentrations of H_2_S donors on sperm motility and viability, and similar to our previous results (Figs. 1 and 2) we observed that these concentrations of H_2_S donors improved both sperm motility and viability at 90 min time point.

As it is depicted in Fig. 3C, the sperm membrane integrity was improved significantly following the treatment with 1.5 µM NaHS (86.67 ± 4.7) and 15 µM GYY4137 (87.4 ± 5.7) compared to control group, while 3 µM NaHS (73.75 ± 9.4) and 30 µM GYY4137 (77.5 ± 2.8) did not improve the membrane integrity to a significant level.

**Figure 4:**
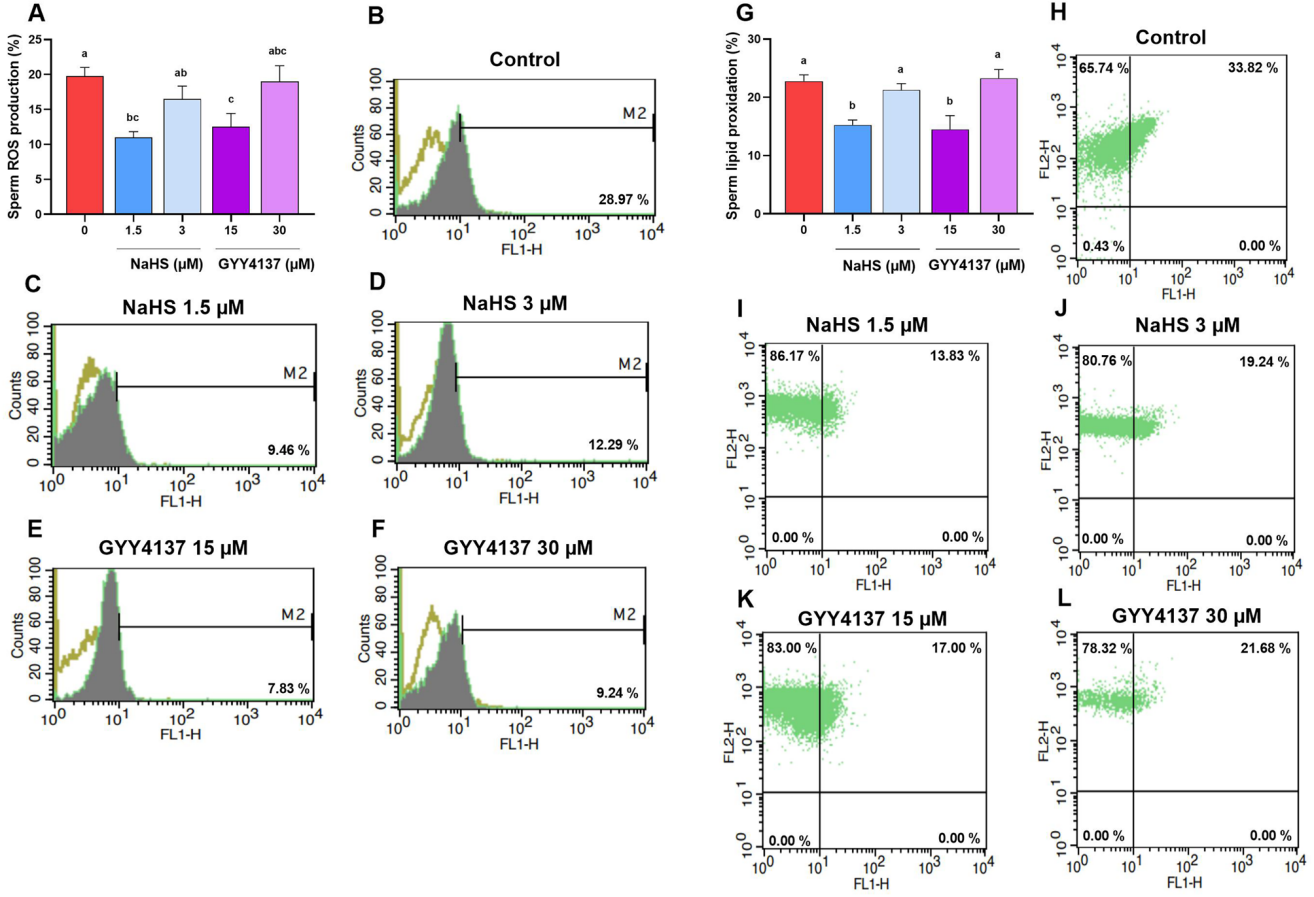
Optimal concentrations of NaHS and GYY4137 decrease ROS production and lipid peroxidation. The effect of optimal concentrations of NaHS (1.5 and 3 µM) and GYY4137 (15 and 30 µM) on (**A**–**F**) sperm ROS production and (**G**–**L**) lipid peroxidation during 1.5 h incubation at 38.5 °C in goat species. Each column represents the mean ± standard error of mean (SEM), and different letters indicate significant differences (*P* < 0.05).

Regarding to the regulatory effect of H_2_S on ROS production in biological systems, in the next step we assessed the level of ROS production following the treatment with the optimal concentrations of H_2_S donors. As it is shown in Fig. 4A, 1.5 µM NaHS (11.2 ± 0.816) and 15 µM GYY4137 (12.5 ± 1.93) significantly lowered the level of ROS compared to control group (19.75 ± 1.25). Interestingly, the highest concentrations of NaHS (3 µM) and GYY4137 (30 µM) (16.5 ± 1.84 and 19.0 ± 2.27, respectively) did not decreased the ROS level compared to control group.

Finally, as a consequence of oxidative stress, we evaluated the lipid peroxidation using BODIPY reagent. As it is revealed in Fig. 4B, similar to the level of ROS production, 1.5 µM NaHS (15.25 ± 085) and 15 µM GYY4137 (14.5 ± 2.36) significantly decreased the level of lipid peroxidation compared to control group (22.75 ± 1.1). Interestingly, again similar to the level of ROS production, the highest concentrations of NaHS and GYY4137 (3 and 30 µM, respectively) (21.25 ± 1.1 and 23.25 ± 1.54, respectively) did not decrease the lipid peroxidation level compared to control group.

### Enhancement of sperm functional parameters after thawing by supplementing the extender with H_2_S donors before cryopreservation

After determining the optimal concentrations of NaHS and GYY4137, we supplemented the extender before cryopreservation with these concentrations to evaluate their effects on different sperm functional parameters after thawing.

**Figure 5:**
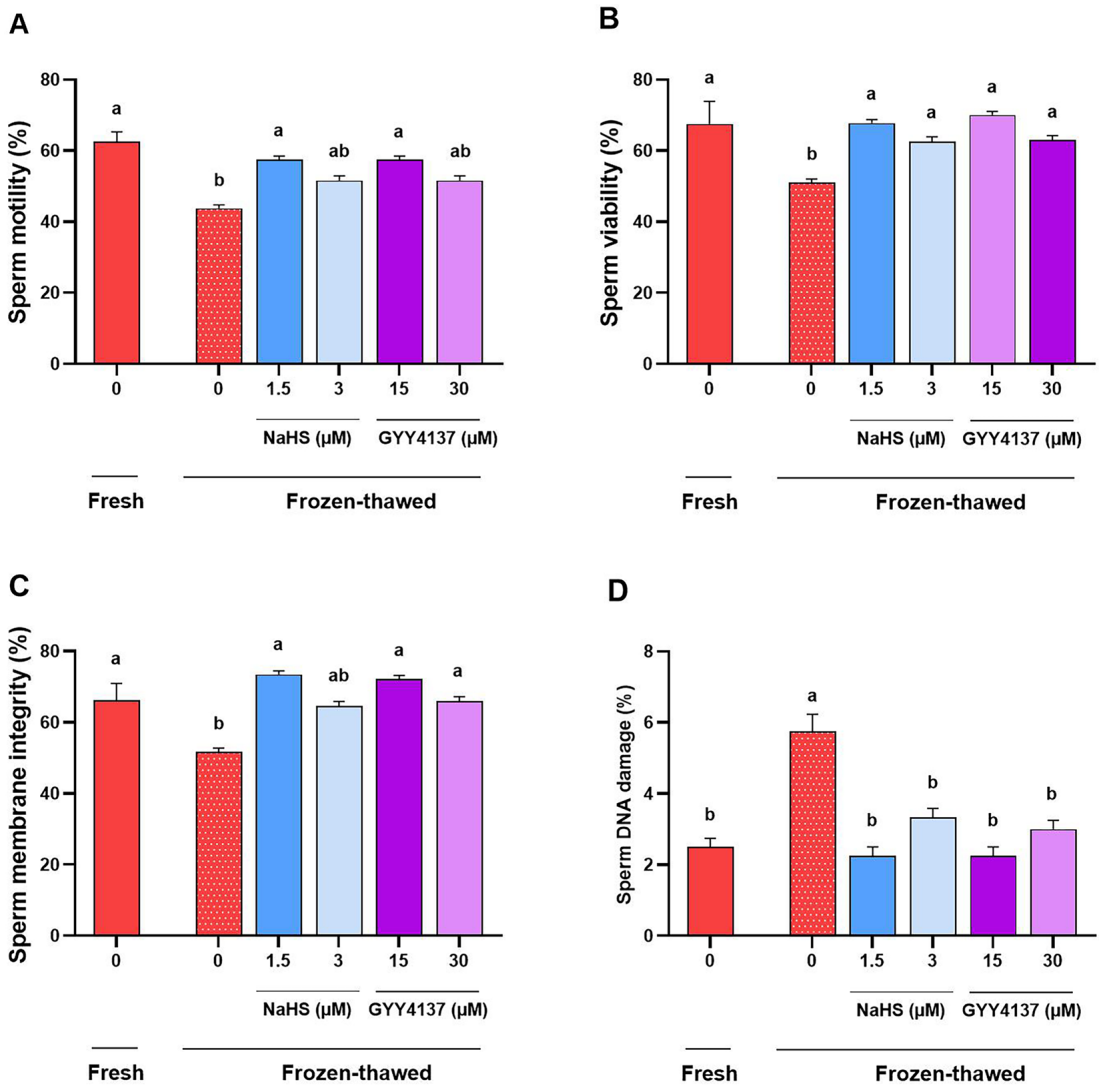
Optimal concentrations of NaHS and GYY4137 enhance functional parameters during cryopreservation. The effect of supplementation of extender with optimal concentrations of NaHS (1.5 and 3 µM) and GYY4137 (15 and 30 µM) during cryopreservation on (**A**) sperm motility, (**B**) viability, (**C**) membrane integrity and (**D**) DNA damage after thawing in goat species. Each column represents the mean ± standard error of mean (SEM), and different letters indicate significant differences (*P* < 0.05).

As shown in Fig. 5A,C, supplementing the extender with 1.5 µM NaHS and 15 µM GYY4137 demonstrated a significant improvement in both sperm motility (57.5 ± 2.5 and 58.7 ± 1.25, respectively) and membrane integrity (73.5 ± 3.12 and 72.25 ± 3.47, respectively) compared to the frozen-thawed control group (sperm motility: 43.75 ± 1.25 and membrane integrity: 51.75 ± 3.11) (*P* < 0.05). These values were statistically similar to those observed in the fresh control group (sperm motility: 62.5 ± 1.44 and membrane integrity 66.25 ± 2.39) (*P* > 0.05). However, 3 µM NaHS and 30 µM GYY4137 did not result in a significant improvement of sperm motility and membrane integrity compared to the frozen-thawed control group (*P* > 0.05) except for 30 µM GYY4137 that significantly improved the membrane integrity (66.0 ± 3.01) compared to the frozen-thawed control group (51.75 ± 3.11) (*P* < 0.05).

Furthermore, we conducted sperm viability and DNA damage assessments following the thawing procedure to evaluate the impact of H_2_S donors. The optimal concentrations of NaHS (1.5 and 3 µM) and GYY4137 (15 and 30 µM) exhibited a significant improvement in sperm viability compared to the frozen-thawed control group (*P* < 0.05), reaching a similar level observed in the fresh control group (*P* > 0.05, Fig. 5B). Moreover, all treated groups demonstrated a significant reduction in sperm DNA damage compared to the frozen-thawed control group (*P* < 0.05), with the values resembling that observed in the fresh control group (*P* > 0.05, Fig. 5D).

### The supplementation of the extender with H_2_S donors before cryopreservation restored the redox state after thawing

**Figure 6:**
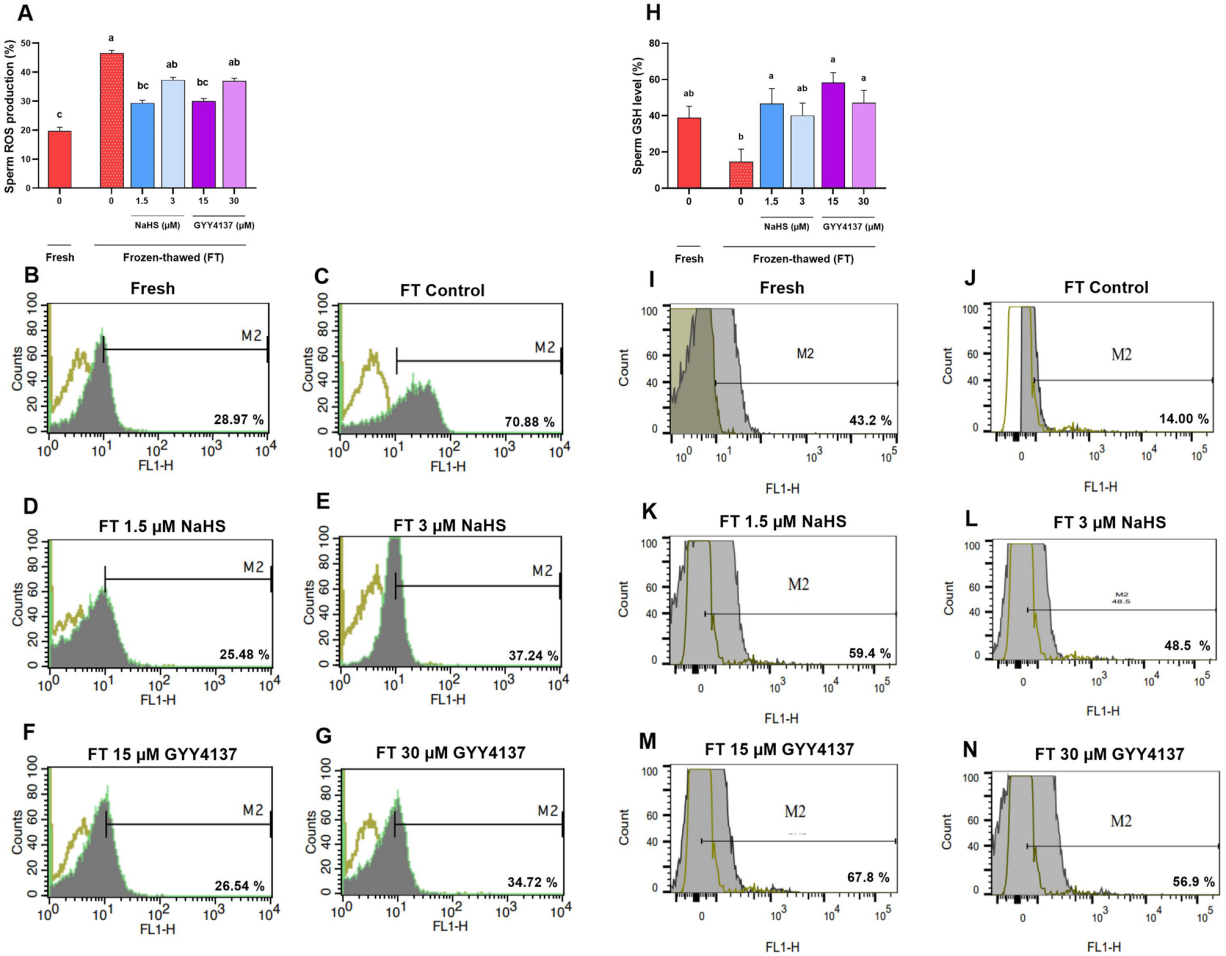
Optimal concentrations of NaHS and GYY4137 improve redox state during cryopreservation. The effect of supplementation of extender with optimal concentrations of NaHS (1.5 and 3 µM) and GYY4137 (15 and 30 µM) during cryopreservation on (**A**–**G**) sperm ROS production and (**H**–**N**) GSH level after thawing in goat species. Each column represents the mean ± standard error of mean (SEM), and different letters indicate significant differences (*P* < 0.05).

Following the thawing procedure, we assessed the ROS and GSH levels to evaluate the effect of H_2_S donors on the redox state. Our data demonstrated that 1.5 µM NaHS and 15 µM GYY4137 significantly reduced the ROS level (29.4 ± 2.18 and 30.20 ± 1.93, respectively) compared to the frozen-thawed control group (46.60 ± 3.26) (Fig. 6A–G, P < 0.05), with the values resembling that observed in the fresh control group (19.75 ± 1.75) (*P* < 0.05, Fig. 5D). Furthermore, as it is depicted in Fig. 6H–N, 1.5 µM NaHS, 15 and 30 µM GYY4137 significantly elevated the GSH level (46.68 ± 8.63, 58.27 ± 5.55 and 47.16 ± 6.79, respectively) compared to the frozen-thawed control group (14.70 ± 6.86) (*P* < 0.05), reaching a similar level observed in the fresh control group (38.90 ± 6.35) (*P* > 0.05, Fig. 6H–N).

**Figure 7:**
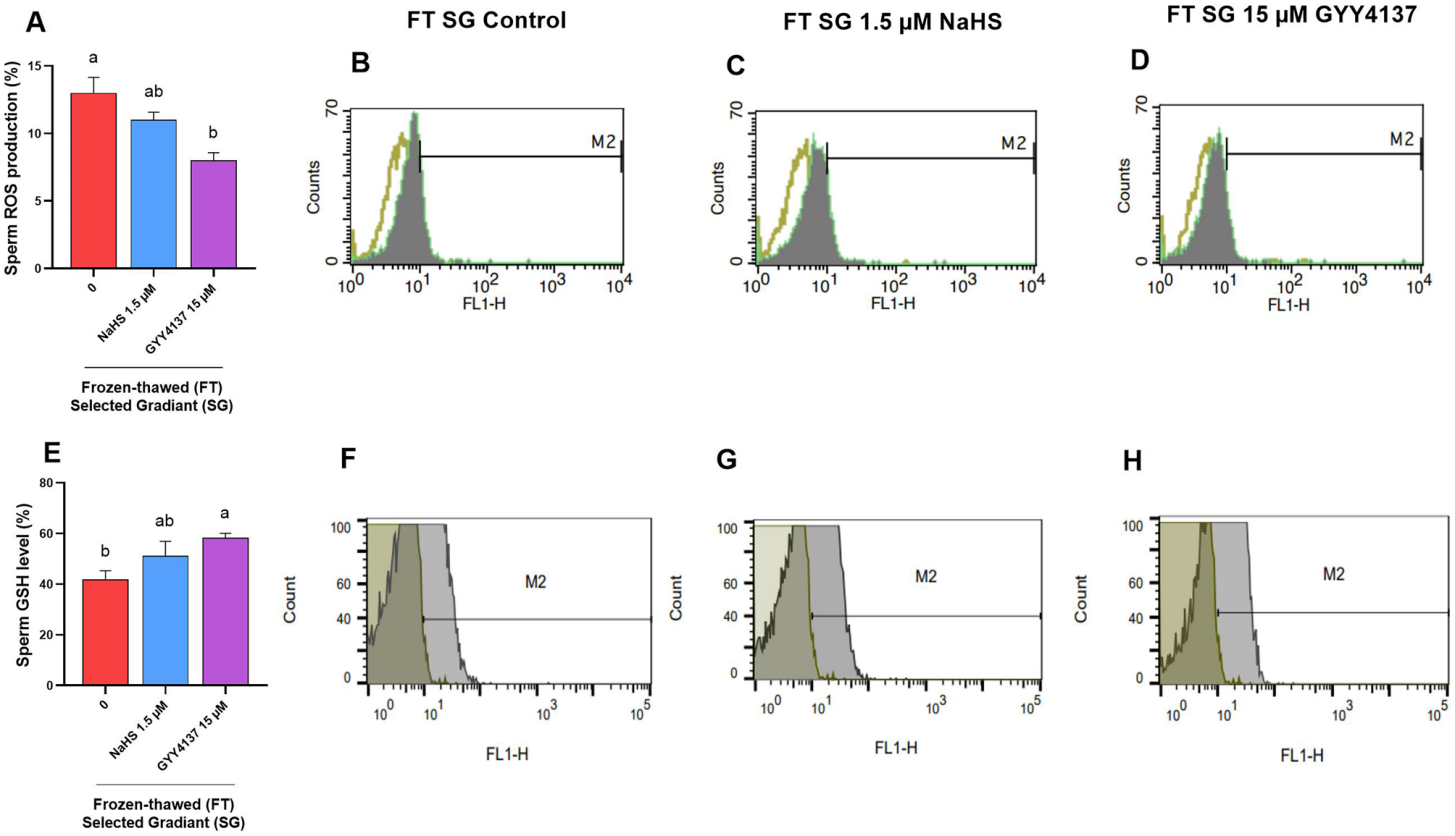
Optimal concentrations of NaHS and GYY4137 improve redox state during cryopreservation in gradient selected sperm. The effect of supplementation of extender with optimal concentrations of NaHS (1.5 and 3 µM) and GYY4137 (15 and 30 µM) during cryopreservation on (**A**–**D**) sperm ROS production and (**E**–**H**) GSH level after thawing in gradient selected sperm. Each column represents the mean ± standard error of mean (SEM), and different letters indicate significant differences (*P* < 0.05).

Additionally, we also assessed the redox state in the treated sperm (1.5 µM NaHS and 15 µM GYY4137) selected post density gradient centrifugation. As it is depicted in Fig. 7A,E, 15 µM GYY4137 significantly reduced the ROS level (8.0 ± 0.557, *P* < 0.05) and increased the GSH level (58.30 ± 1.80, P < 0.05) compared to the control group (ROS level: 13.0 ± 1.154 and GSH level: 49.94 ± 3.37, respectively). However, 1.5 µM NaHS did not restore the redox state in gradient selected sperm compared to the control group (Fig. 7, *P* > 0.05).

### The supplementation of the extender with H_2_S donors before cryopreservation enhanced the lipid peroxidation and mitochondrial membrane potential after thawing

Since we observed that H_2_S donors are effective on preserving sperm motility and restoring redox balance, subsequently, we evaluated the lipid peroxidation and mitochondrial membrane potential.

**Figure 8:**
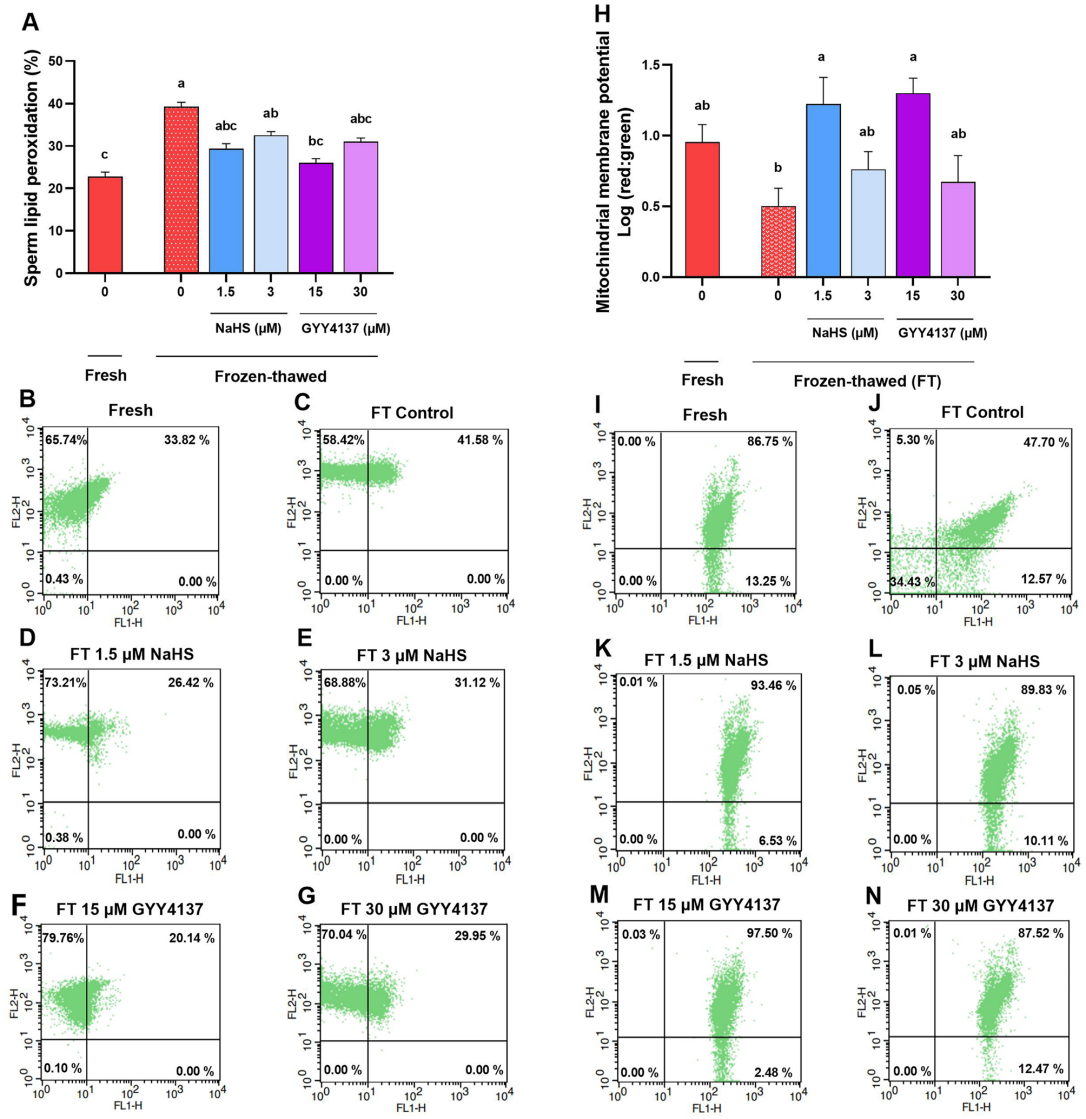
Optimal concentrations of NaHS and GYY4137 reduce lipid peroxidation and enhance mitochondrial membrane potential during cryopreservation. The effect of supplementation of extender with optimal concentrations of NaHS (1.5 and 3 µM) and GYY4137 (15 and 30 µM) during cryopreservation on (**A**–**G**) sperm lipid peroxidation and (**H**–**N**) mitochondrial membrane potential after thawing in goat species. Each column represents the mean ± standard error of mean (SEM), and different letters indicate significant differences (*P* < 0.05).

Among the optimal concentration of NaHS and GYY4137, we observed that only 15 µM GYY4137 significantly decreased the lipid peroxidation (26.0 ± 2.41) compared to the frozen-thawed control group (38.0 ± 2.12) (Figs. 8A–G, P < 0.05) that was similar to the fresh control group (22.75 ± 1.10) (*P* > 0.05).

Following the evaluation of lipid peroxidation in thawed sperm, we also assessed the mitochondrial membrane potential using JC1 reagent. As it is demonstrated in Fig. 8H–N, we observed that supplementing the extender with 1.5 µM NaHS and 15 µM GYY4137 significantly improved mitochondrial membrane potential (1.00 ± 0.19 and 1.30 ± 0.11, respectively, *P* < 0.05) compared to the frozen-thawed control group (0.50 ± 0.12). These values were statistically similar to that observed in the fresh control group (0.83 ± 0.04, *P* > 0.05).

Altogether, our results reveal that H_2_S donors, exclusively 1.5 µM NaHS and 15 µM GYY4137, could restore different sperm functional parameters which can enhance their quality during fertilization procedure and increase the efficiency of IVF.

### The supplementation of the extender with H_2_S donors before cryopreservation enhanced the in vitro development and the quality of embryos

The effect of selected optimal concentrations of H_2_S donors including 1.5 µM NaHS and 15 µM GYY4137 on the pre-implantation embryo development is presented in Table 1. Firstly, no difference was recorded in cleavage rates among the experimental groups (*P* > 0.05). But, the matured COCs inseminated with the sperm frozen in the extender supplemented by either 1.5 µM NaHS or 15 µM GYY4137 showed significantly (*P* < 0.05) higher blastocyst rates (43.02 ± 2.59 and 48.34 ± 0.47, respectively) compared to the control group (32.29 ± 2.96). In addition, the hatching rate was significantly higher in 15 µM GYY4137 (54.27 ± 2.69, *P* < 0.05) but not in 1.5 µM NaHS (46.70 ± 1.88, *P* > 0.05) compared to the control group (39.58 ± 2.86).

**Table 1:** Comparison between cleavage, blastocyst, and hatching/hatched rates in embryos derived from frozen/thawed goat sperm with 1.5 µM NaHS and 15 µM GYY4137. The data are presented as mean ± standard error of mean (SEM), and different letters indicate significant differences (*P* < 0.05).

Groups	No. of replications	No. of presumptive zygotes	Cleavage rate % (no. of cleaved embryos)	Blastocyst rate/total zygotes % (no. of blastocysts)	Hatching rate % (no. of hatching/hatched blastocysts)
Control	3	141	83.21 ± 1.74 (128)^a^	32.29 ± 2.96 (50)^a^	39.58 ± 2.86 (20)^a^
GYY4137 15 µM	3	168	87.86 ± 1.32 (159)^a^	48.34 ± 0.47 (87)^b^	54.27 ± 2.69 (47)^b^
NaHS 1.5 µM	3	171	87.37 ± 1.40 (162)^a^	43.02 ± 2.59 (79)^b^	46.70 ± 1.88 (37)^ab^

**Figure 9:**
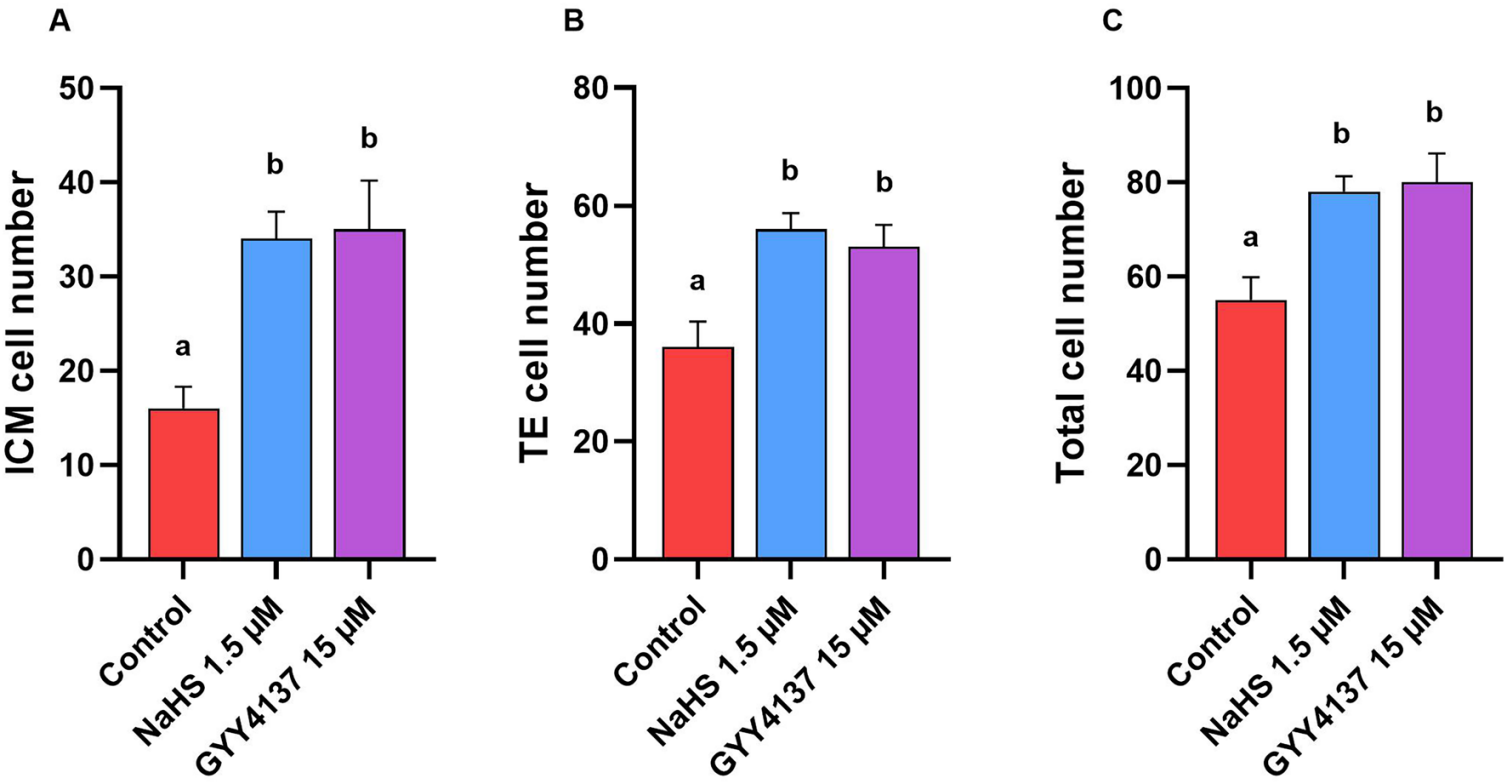
Optimal concentrations of NaHS and GYY4137 improve the blastomere allocations in derived blastocysts. Comparison between (**A**) inner cell mass (ICM), (**B**) trophectoderm cells TE, and (**C**) total cell number (TCN) in blastocysts derived from frozen/thawed goat sperm supplemented with 1.5 µM NaHS and 15 µM GYY4137. Each column represents the mean ± standard error of mean (SEM), and different letters indicate significant differences (*P* < 0.05).

Subsequently, we assessed the quality of the derived blastocysts in terms of the blastomere allocation into ICM and TE and the TCN of blastocysts. As observed in Fig. 9A, there was a higher ICM number in the 1.5 µM NaHS and 15 µM GYY4137 groups (31.4 ± 2.93 and 35.44 ± 5.23, respectively) compared to the control group (16.80 ± 2.33, *P* < 0.05, Fig. 8A). Furthermore, the TE number was significantly higher in 1.5 µM NaHS and 15 µM GYY4137 groups (53.50 ± 4.47 and 56.07 ± 3.74, respectively) compared to the control group (36.0 ± 4.38, *P* < 0.05, Fig. 9B). Finally, the TCN was significantly higher in 1.5 µM NaHS and 15 µM GYY4137 groups (80.69 ± 3.31 and 78.14 ± 6.12, respectively) compared to the control group (55.25 ± 4.92, *P* < 0.05, Fig. 9C).

## Discussion

Sperm cryopreservation is a valuable technique used in assisted reproductive technologies (ARTs) for both humans and farm animals. It allows for the long-term storage of sperm samples, which can be utilized in future for ART procedures. While the freezing technique is generally considered safe, exposure to cold temperatures could potentially cause biochemical and oxidative damage to the structure of sperm. This damage could lead to impairment in various functional parameters, ultimately diminishing the fertilization potential of frozen-thawed sperm. One potential approach to alleviate these detrimental effects is to incorporate antioxidants into the sperm freezing media or the extender. Numerous studies have explored the supplementation of various antioxidants into the extender as a conventional strategy during sperm freezing to counteract cryoinjury. However, there is significant interest in utilizing physiological antioxidants that closely mimic the in vivo microenvironment.

H_2_S (hydrogen sulfide), as the third and recently discovered gasotransmitter, plays a crucial role in the physiology and pathophysiology of various cell types under oxidative stress. Due to numerous studies highlighting anti-oxidative, anti-inflammatory, and anti-apoptotic properties of H_2_S, we have prompted to investigate its potential application in sperm cryopreservation.

In this study, up to our knowledge, for the first time, we supplemented the sperm extender with two different H_2_S donors, NaHS as a fast-releasing donor and GYY4137 as a slow releasing donor, during freezing process to assess their cryogenic protection on various sperm functional parameters in goat species. The results showed that (1) 1.5 and 3 µM NaHS, and 15 and 30 µM GYY4137 more efficiently preserved different sperm functional parameters than other concentrations compared to the control group during in vitro incubation at 38.5 °C, (2) The supplementation of the extender with optimal concentrations of both NaHS and GYY4137 improved various function parameters of frozen-thawed sperm compared to the control, (3) Finally, our data revealed that 1.5 µM NaHS and 15 µM GYY4137 significantly improved both blastocyst formation rate and quality of blastocysts compared to the control group.

One of the primary characteristics of frozen-thawed sperm is their imbalanced redox state that mainly is manifesting as oxidative stress. Oxidative stress results from an imbalance between reactive oxygen species (ROS) and antioxidants and is a significant concern in sperm cryopreservation. Numerous studies have investigated the effects of various antioxidants, such as alpha-lipoic acid, coenzyme Q10, folate, inositol, selenium, zinc, and other vitamins on sperm functional parameters. These studies have demonstrated a remarkable improvement in the viability and quality of frozen-thawed sperm in different species^51,52^.

Our results revealed that optimized concentrations of both NaHS and GYY4137 reduced the ROS levels and elevated the GSH levels in frozen-thawed sperm. Our data supports the previous findings in different cells and tissues such as gastric cells (14), lungs (15, 26), neurons (13, 25), testicular germ cells (4) and hepatic tissue (18). While, in the mentioned studies, oxidative stress was induced by in vivo or in vitro ROS-generating systems, in the current study, we used freezing process as a model to induce oxidative stress damage in goat sperm. Hydrogen sulfide (H_2_S) possesses potent antioxidant properties and can effectively counteract superoxide anions^53^ and scavenge other harmful reactive oxygen species (ROS), thereby mitigating oxidative stress and preserving cellular integrity. The sulfide similar to other small thiols such as cysteine and GSH may act as a redox-controlling molecule^54,55^. Kimura and colleagues found that H_2_S increases endogenous GSH levels by enhancing gamma-glutamylcysteine synthetase activity and upregulating cystine transport and protects neurons from oxidative stress^54^. In addition, Lu and colleagues reported that NaHS induces glutamate uptake and increases cysteine transport and GSH synthesis^56^. In addition, H_2_S can regulate the activity of various antioxidant enzymes, including superoxide dismutase (SOD), catalase, and glutathione peroxidase^40^. These enzymes are responsible for detoxifying ROS and maintaining cellular redox balance. H_2_S can enhance the activity of these enzymes, leading to increased antioxidant capacity. However, we did not assess the activity of these related enzymes in the current study in treated samples and it remained to be elucidated.

Previous studies^22,57^ mostly used a single H_2_S donor in sperm samples under oxidative stress conditions. However, there are few studies that used both a fast- (e.g., NaHS or Na2S) and a slow-releasing donor (e.g., GYY4137) in human^23^ and porcine^58^ spermatozoa. In this study we employed and compared two H_2_S-releasing agents, one fast (NaHS) and one slow (GYY4137) releasing donors in sperm cryopreservation. In this current study, we found that despite to GYY4137, NaHS at lower concentrations (< 300 µM) preserves both sperm motility and viability during incubation at 38.5 °C throughout all time points up to 270 min and in the thawed sperm, whereas, NaHS at higher concentrations (300 and 600 µM) show cytotoxic effects on goat sperm. These results may exhibit the well-known biphasic biological dose–response of H_2_S (29): while at lower concentrations they act as antioxidants and reduce oxidative stress and demonstrate the cytoprotective effects, at higher concentrations by scavenging excessive amounts of ROS they imbalanced the redox state and induce their cytotoxic effects on goat sperm. An alternative explanation is that high concentrations of H_2_S may tilt the balance toward reductive stress. It has been stated that both "oxidative" and "reductive" stress can increase the ROS levels^57,58^. So, we can conclude that this delicate balance helps maintain cellular redox homeostasis and prevents oxidative damage during cryopreservation. In addition to this explanation, Zhao et al. found that the Na_2_S upregulates the adenosine 5′-monophosphate-activated protein kinase pathway and downregulates Na^+^/K^+^ ATPase activity and protein kinase B pathway and through modulating these pathways exerts its inhibitory effects on sperm motility^59^. However, another study demonstrated that that H_2_S promotes the secretion of K^+^ in the epididymis and suppresses the sperm motility^28^. Based on previous studies, another probable explanation for the reduced sperm motility caused by NaHS and GYY4137 (at the highest concentrations) can be related to the inhibitory effect of H_2_S on cytochrome C oxidase (complex IV)^60,61^.

In line with our studies, Wang and Meng reported that in infertile men, especially in patients with asthenospermia, the concentration of H_2_S in the seminal plasma and the expression of CBS in the sperm have decreased. Supplying exogenous H_2_S to seminal fluid improved sperm motility in these patients. In addition, in animal models of CBS-deficiency that have defects in H_2_S production, a decrease in sperm motility was reported. Furthermore, the study by Wang et al. showed that the decrease in production of endogenous H_2_S and downregulation of CBS expression are associated with impaired spermatogenesis and a blood–testis barrier. These studies demonstrated that compensation of H_2_S production or expression of CBS could mitigate spermatogenic failure^23^. In addition, in a study by Pintus et al., they reported that NaHS and GYY4137, two H_2_S donors, maintained sperm motility and reduced acrosome loss under oxidative challenges^58^. Furthermore, The same group of authors recently reported another hydrogen sulfide donor, amino acid-derived NTAs), increased progressive sperm motility in short-term incubation under oxidative stress^22^.

Interestingly, we also found that optimized concentrations of both NaHS (1.5 and 3 µM) and GYY4137 (15 and 30 µM), in addition to improving sperm motility and viability, enhanced membrane integrity, and lipid peroxidation in both in vitro conditions at 38.5 °C and in frozen-thawed sperm. Furthermore, the optimized concentrations of both donors reduced the DNA damage in frozen-thawed sperm. During the cryopreservation process, cells are exposed to various stressors such as oxidative damage that can trigger apoptosis, a programmed cell death mechanism. In this study, we have shown that both H_2_S donors modulate the redox state in treated sperm. So, we explain that maybe H_2_S alleviates the DNA damage by diminishing the oxidative stress and modulating the redox state. In addition, hydrogen sulfide has been shown to mitigate apoptosis by regulating apoptotic signaling pathways and preserving the functionality of mitochondria, the powerhouse of the cell. In this study, we also have shown the improvement in mitochondrial function by assessing the mitochondrial membrane potential. In a study by Ning et al. it has been shown that application of NaHS improved mitochondrial function characterized by increased ATP production, O_2_ consumption, and decreased ROS production. The results also revealed that H_2_S may prevent cells from apoptosis pathway^27^. In addition, in another study, Li and Bian measured the expression of several proteins of the mitochondrial apoptotic pathway: Bax, Bcl-2, and caspase 3. In this study, they demonstrated that application of GYY4137 reduced the expression of Bax and caspase 3 in heat-exposed testis cells and preserved the expression of Bcl-2 compared to the untreated group^62^.

Several studies in human and animal models show that oxidative stress in sperm and DNA damage can have negative effects on embryo development^63^. In this study, we showed that treatment of frozen sperm with 1.5 µM NaHS and 15 µM GYY4137 significantly improved blastocyst rate and quality of blastocysts compared to the control group. As we know, during cryopreservation, exposure of sperm to various stresses, including oxidative stress could induce oxidative damage to the DNA, proteins, and cell membranes. This oxidative damage can negatively affect the ability of sperm to fertilize the oocyte. In the current study, by supplementing the extender with H_2_S donors, we found that H_2_S can neutralize ROS and protect cells from oxidative damage. By reducing oxidative stress, these donors help maintain the integrity and functionality of sperm cells during the freezing process, as we have shown in this study. So, H_2_S can enhance the sperm ability to fertilize oocytes, leading to higher preimplantation developmental rate and quality of derived blastocyst. This approach can also be beneficial in assisted reproductive technologies, particularly in cases where sperm quality with cryopreservation techniques are a concern.

## Conclusion

In conclusion, in this study, we supplemented the sperm extender with two H_2_S donors, NaHS and GYY4137, before the freezing process in goat sperm. The results showed that optimal concentrations of both NaHS and GYY4137 effectively preserved various sperm functional parameters during both in vitro incubation at 38.5 °C and before freezing process. In this study we showed that H_2_S donors improved sperm motility, viability, and membrane integrity, compared to the control group. These donors by producing exogenous H_2_S scavenge harmful ROS and mitigate oxidative stress. In addition, these donors improve mitochondrial function and protect against DNA damage. Finally, these donors enhanced the blastocyst formation rate and quality of derived embryos. The findings from this study revealed that use of physiological antioxidants like H_2_S donors in sperm cryopreservation can help maintain cellular redox homeostasis, prevent oxidative damage, and enhance the fertilization potential of frozen-thawed sperm. Overall, the supplementation of sperm extender with H_2_S donors, during the freezing process can be a promising strategy to improve the success rates of ART procedures and enhance preimplantation developmental outcomes in assisted reproductive technologies**.**

## Materials and methods

### Ethics

This study is in accordance with the ARRIVE guidelines 2.0. All animal care and procedures were approved by the Institutional Ethical Committee of the Royan Institute (IR.ACECR.AEC.1401.023). In addition, all methods used in the current study were carried out under the Institutional Review Board and Institutional Ethical Committee of the Royan Institute guidelines and regulations.

### Chemicals

Unless otherwise mentioned, all materials and media in this study were obtained from Sigma-Aldrich Company (St Louis, MO, USA) and Gibco (Life Technologies, Rockville, MD, USA), respectively. The AndroMed^®^ One-step medium (REF: 13503) was obtained from Minitube company.

### Preparation of NaHS and GYY4137 solutions

Commercially available sodium hydrosulfide hydrate as dissolved in distilled water (161527, Sigma) and then formulated to 45 mM stock solution and used freshly in each replication. The desired working solutions including, 0.3, 0.75, 1.5, 3, 30, 300 and 600 μM, were prepared by diluting the stock solution with AndroMed^®^. Furthermore, commercially available GYY4137 dichloromethane complex (SML0100; Sigma) was dissolved in DMSO and then formulated to 12 mM stock solution and stored at − 20 °C. AndroMed^®^ was used to dilute the stock solution to obtain the desired concentrations (3, 7.5, 15, 30, 300 and 600 µM) later. The control group in GYY4137 groups was treated with DMSO similar to the concentration of DMSO that was in 600 µM GYY4137 group. However, to present the name of this group in an abbreviated form we only mentioned control group.

### Semen collection and processing

Semen samples from five Sannen goats (2–3-year-old), with previously proven fertility, were collected with the aid of an artificial vagina twice to three times a week during the breeding season (September/October 2022). Only ejaculates with more than 1 mL in volume and > 80% motility and normal morphology were entered the experimental design. Semen samples from four to five goats were mixed to exclude individual differences.

### Experimental design

The experimental design of this study included two experiments:

Experiment 1: In this experiment for optimizing the concentration of NaHS and GYY4137, each combined sample was diluted 2 times using AndroMed^®^ containing no NaHS or GYY4137 (control group), 0.3, 0.75, 1.5, 3, 30, 300 and 600 µM of NaHS or 3, 7.5, 15, 30, 300 and 600 µM of GYY4137. The final concentration of sperm in each group was approximately 1.25 × 10^8^ sperm/mL. After incubating the samples in open vials for 90, 150, 210 and 270 min at 38.5 °C in incubator (CO_2_/O_2_-regulated incubator set at 20% O_2_ and 5% CO_2_), the motility and viability were assessed to determine the optimal concentrations of NaHS and GYY4137. After selecting the optimal concentrations of NaHS and GYY4137, further assessments including membrane integrity, lipid peroxidation, ROS production were evaluated at 90 min time-point.

Experiment 2: in this experiment the optimized concentrations of NaHS (1.5 and 3 µM) and GYY4137 (15 and 30 µM) from experiment 1 were used for cryopreservation procedure. To this aim, the diluted semen was chilled to 4 °C in 1 h gradually and then drawn into 0.5 mL straws. Subsequently, the straws were balanced at 0 °C for 20 min and then were exposed to liquid nitrogen (LN_2_) vapor (the distance was 4 cm) for 12 min, plunged into LN_2_, and stored until the time of use. Approximately, one week later the straws were removed from the LN_2_ and thawed by immersing in a water bath at 37 °C for 45 s. Following thawing, different parameters including motility, viability, membrane integrity, DNA integrity, lipid peroxidation, ROS and GSH levels, and mitochondrial membrane potential were evaluated. Finally, the fertilization ability of cryopreserved sperm samples was also assessed.

### Sperm motility and viability

We acknowledge the importance of CASA as a precise method for sperm motility assessment but due to resource constraints, we were unable to utilize CASA for sperm motility analysis and sperm motility was subjectively assessed in this study. The motility of sperm was evaluated by placing 1 × 10^6^ sperm/mL of diluted sperm on pre-warmed slides at 37.5 °C, and determining the number of actively swimming sperm using 10× and 20× objective lenses and Olympus CKX41SF inverted phase contrast microscope. Each aliquot volume used for assessment was 20 µL. At least 200 sperm were analyzed on each slide for motility^64^. To ensure consistency, we performed multiple evaluations by three observers and averaged the results.

Sperm viability was assessed using eosin-nigrosine staining method. A drop of sperm (1 × 10^6^ sperm/mL) was mixed with a dye solution consisting of 5% red eosin solution and 10% blue nigrosine solution. The mixture was then spread on a smear slide and air-dried at room temperature (RT). The slides were examined under a bright-field light microscope at 1000× magnification using oil immersion. At least 200 sperm were counted on each slide, and the percentage of stained (indicating dead) and unstained (indicating live) sperm was calculated^65^.

### Sperm plasma membrane integrity

The normal osmotic pressure for animal sperm is 425 mOsm/L. Therefore, when sperm exposed to a low-osmolality environment, they exhibit a rapid response characterized by tail swelling. Healthy sperm with intact plasma membrane respond to the solution, while unhealthy sperm remain unresponsive. In this test, sperm with coiled tails were considered alive, whereas sperm with straight tails were deemed nonviable. The procedure involved mixing 1 × 10^6^ sperm/mL with 90 µL of hypoosmotic swelling test solution (HOST). Sodium citrate and fructose-based solutions with 125 mOsm/L were used for HOST test. HOST medium, followed by a 30 min incubation period at 38.5 °C. After incubation, a 10 µL drop of the sample was prepared and observed under a light microscope on a slide at 200× magnification. At least 200 sperm were counted in each treatment group, and the percentage of sperm with intact membranes was calculated^66,67^.

### Sperm ROS levels

Intracellular H_2_O_2_ levels in the sperm were assessed using H2DCFDA staining. H2DCFDA as a cell-permeable dye has the affinity to localize in the hydrophobic regions of the cell. H2DCFDA is oxidized by intracellular ROS into dichlorofluorescein (DCF) and emits fluorescence at 530 nm in response to 488 nm excitation. Approximately, 1 × 10^6^ sperm/mL in phosphate buffer saline without calcium and magnesium (PBS^−^) were stained with H2DCFDA (1 µM) and incubated for 30 min at 37 °C. The samples were then analyzed using a FACSCalibur flow cytometer (Becton Dickinson) within 1 h after preparation^68,69^.

### Glutathione levels

Sperm GSH levels were measured with CellTracker Blue™ CMF2HC Molecular Probe (Invitrogen Inc., Carlsbad, CA, USA). Approximately, 1 × 10^6^ sperm/mL in phosphate buffer saline without calcium and magnesium (PBS^−^) were stained with CellTracker Blue dye (10 μM) and incubated for 30 min at 37 °C. The samples were then analyzed with BD FACSLyric™ Flow Cytometry System^1^.

### Lipid peroxidation

Lipid peroxidation was measured using BODIPY™ 581/591 C11 (Lipid Peroxidation Sensor) (Invitrogen Inc., Carlsbad, CA, USA; D3861). Approximately, a suspension of 1 × 10^6^ sperm/mL were stained with BODIPY™ 581/591 C11 (5 mM) for 30 min at 37 °C. Subsequently, the samples were then analyzed with BD FACSLyric™ Flow Cytometry System^70^.

### Sperm mitochondrial activity

The fluorescent dye JC-1, a cationic dye that accumulates in energetic mitochondria, was used to investigate sperm mitochondrial activity. The sperm sample with a concentration of 2 × 10^6^/mL was stained with a dye concentration of 8 µM (56) and incubated for 30 min at RT. The flow cytometry device was set with a FCCP positive control with a concentration of 50 µM for 60 min. The approximate excitation peak is 488 nm, and the approximate emission peaks of the monomeric and aggregated forms are 529 nm and 590 nm, respectively. Two types of JC-1 are present in stained mitochondrial plasma. One is monomeric, which emits green fluorescence at low ΔΨm, and the other is aggregated, emitting red fluorescence at high ΔΨm. The Δψm of sperm in each treatment group was calculated as the logarithm of the ratio of red (aggregated) to green (monomeric) fluorescence (Becton Dickinson). The analysis was performed in quadruplicate (n = 4) (1).

### DNA damage analysis

Acridine orange (AO) is a DNA‐intercalating dye used for detection of DNA damage in sperm. In brief, the air-dried smears were fixed in Conroy’s fixative (methanol: acetic acid: 3:1 (v/v)) at 4 °C for 2 h. Then, the slides were allowed to dry at RT. Then, the slides were stained with AO solution (1 mg/mL) for 10 min at RT. Subsequently, the slides were rinsed in distilled water. Subsequently, at least 200 sperm were analyzed using a fluorescent microscope (Olympus BX51) at 1000× magnification. AO emits green fluorescence when binds to double stranded DNA and red fluorescence when binds to single stranded DNA (ss-DNA). Finally, the number of sperm with red fluorescence to total number of analyzed sperm was calculated and presented as percentage to demonstrated the percentage of DNA damage^71,72^.

### Oocyte retrieval and in vitro maturation (IVM)

The goat ovaries were collected from a local slaughterhouse and transferred to the laboratory within 1 h. After trimming the ovaries and separating the surrounded tissues, the ovaries were kept in the normal saline supplemented with penicillin/streptomycin at 15 °C for an overnight.

In the following day, the ovaries were washed extensively and the cumulus–oocyte complexes (COCs) were recovered from follicles with diameter of 2–6 mm with a 21-gauge needle attached to a vacuum pump with adjusted pressure (60–70 mmHg). The aspiration medium was the HEPES-buffered tissue culture medium 199 (TCM199; HTCM199) supplemented with 10% fetal bovine serum (FBS) and heparin (10 μL/mL). After transferring the aspirated medium to a culture dish, the medium was searched for COCs with homogenous cytoplasm and at least three compact cumulus cells for IVM^42^. After washing the selected COCs in washing medium, they were randomly cultured in a defined maturation medium in groups of 10 in 50 μL droplets^41,43^. The COCs were incubated for 20 h at 38.5 °C in a humidified atmosphere containing 6.5% CO_2_ under mineral oil.

### Sperm preparation and IVF

The IVF procedure was carried out similar to our previous studies^73,74^. Untreated and treated frozen-thawed Sannen goat semen (with either 1.5 µM NaHS or 15 µM GYY4137) were used for IVF procedure. The swim down method was used for isolation of motile sperm from immotile sperm. Subsequently, 10 matured COCs were co-incubated with 1 × 10^6^/mL isolated motile sperm in droplets of fertilization medium at 38.5 °C, 6.5% CO_2_ and humidified atmosphere under mineral oil. Eighteen hours post insemination, the cumulus cells were removed from presumptive zygotes by pipetting. Thereafter, the presumptive zygotes were cultured in synthetic oviduct fluid (SOF) supplemented with ITS (insulin, transferrin, and selenium) and myo-inositol without glucose and serum for 3 days at 38.5 °C, 6.5% CO_2_ and 5% O_2_ in humidified air under mineral oil. At Day 3, the cleavage rate was assessed and the cleaved embryos were transferred to SOF medium supplemented with charcoal stripped serum. Finally, the blastocyst and hatching rates were evaluated at Day 7 and 8, respectively.

### Differential staining

The hatching/hatched blastocysts from all groups were transferred into PBS^−^ + PVA (poly vinyl alcohol) medium and washed extensively to remove the culture medium. Subsequently, the blastocysts were permeabilized with 0.2% Triton X-100 for 20 s and then transferred to 30 μg/mL propidium iodide (PI) (Sigma-Aldrich) for 45 s. After washing with HTCM + 3 mg/mL bovine serum albumin (BSA), blastocysts were transferred in chilled absolute ethanol supplemented with 10 μg/mL Hoechst 33342 (Sigma-Aldrich) for 20 min. The stained blastocysts were placed on glass slides in mounting fluid and covered with coverslip. The stained blastocysts were captured using an epifluorescence microscope equipped with appropriate fluorescence filters. The inner cell mass (ICM) and the trophectoderm (TE) appeared pink and blue under UV light, respectively.

### Statistical analysis

In this study, all assessments were performed at least three times. The normality of data was checked by the Shapiro–Wilk test. All results were presented in mean ± standard error. One-way analysis of variance (ANOVA) was performed to investigate the effect of the treatments on functional parameters of sperm and cleavage rate and blastocyst (α = 0.05), followed by Tukey's post hoc test by SPSS software (SPSS Science, Chicago, IL, USA). A *P* value < 0.05 was considered statistically significant. GraphPad Prism statistical software package version 8 (GraphPad Software) was used for graph design.

## Data Availability

The datasets used and/or analyzed during the current study available from the corresponding author on reasonable request.

## References

[1] Zhu Z (2019). Resveratrol improves Boar sperm quality via 5AMP-activated protein kinase activation during cryopreservation. Oxid. Med. Cell. Longev..

[2] Khan IM (2021). Impact of cryopreservation on spermatozoa freeze-thawed traits and relevance omics to assess sperm cryo-tolerance in farm animals. Front. Vet. Sci..

[3] Tamburrino L (2023). Cryopreservation of human spermatozoa: Functional, molecular and clinical aspects. Int. J. Mol. Sci..

[4] Sharafi M, Borghei-Rad SM, Hezavehei M, Shahverdi A, Benson JD (2022). Cryopreservation of semen in domestic animals: A review of current challenges, applications, and prospective strategies. Animals.

[5] Hungerford A, Bakos HW, Aitken RJ (2022). Sperm cryopreservation: Current status and future developments. Reprod. Fertil. Dev..

[6] John Aitken R, Clarkson JS, Fishel S (1989). Generation of reactive oxygen species, lipid peroxidation, and human sperm function. Biol. Reprod..

[7] Agarwal A, Saleh RA, Bedaiwy MA (2003). Role of reactive oxygen species in the pathophysiology of human reproduction. Fertil. Steril..

[8] Aitken RJ, Gibb Z, Baker MA, Drevet J, Gharagozloo P (2016). Causes and consequences of oxidative stress in spermatozoa. Reprod. Fertil. Dev..

[9] Siracusa R (2022). NO, CO and H2S: A trinacrium of bioactive gases in the brain. Biochem. Pharmacol..

[10] Majid ASA, Majid AMSA, Yin ZQ, Ji D (2013). Slow regulated release of H_2_S inhibits oxidative stress induced cell death by influencing certain key signaling molecules. Neurochem. Res..

[11] Tyagi N (2009). H2S protects against methionine-induced oxidative stress in brain endothelial cells. Antioxid. Redox Signal..

[12] Kesherwani V, Nelson K, Agrawal S (2013). Effect of sodium hydrosulphide after acute compression injury of spinal cord. Brain Res..

[13] Huang C (2013). Cardioprotective effects of a novel hydrogen sulfide agent–controlled release formulation of s-propargyl-cysteine on heart failure rats and molecular mechanisms. PLoS One.

[14] Sojitra B (2012). Nitric oxide synthase inhibition abrogates hydrogen sulfide-induced cardioprotection in mice. Mol. Cell. Biochem..

[15] Wang C, Wang H-Y, Liu Z-W, Fu Y, Zhao B (2011). Effect of endogenous hydrogen sulfide on oxidative stress in oleic acid-induced acute lung injury in rats. Chin. Med. J..

[16] Otunctemur A (2014). Protective effect of hydrogen sulfide on gentamicin-induced renal injury. Renal Fail..

[17] Sen U (2009). Hydrogen sulfide ameliorates hyperhomocysteinemia-associated chronic renal failure. Am. J. Physiol. Renal Physiol..

[18] Jha S, Calvert JW, Duranski MR, Ramachandran A, Lefer DJ (2008). Hydrogen sulfide attenuates hepatic ischemia-reperfusion injury: Role of antioxidant and antiapoptotic signaling. Am. J. Physiol. Heart Circ. Physiol..

[19] Guo C, Liang F, Masood WS, Yan X (2014). Hydrogen sulfide protected gastric epithelial cell from ischemia/reperfusion injury by Keap1 s-sulfhydration, MAPK dependent anti-apoptosis and NF-κB dependent anti-inflammation pathway. Eur. J. Pharmacol..

[20] Cui J (2013). Protective effect of endogenous hydrogen sulfide against oxidative stress in gastric ischemia-reperfusion injury. Exp. Ther. Med..

[21] Řimnáčová H (2022). Evidence of endogenously produced hydrogen sulfide (H_2_S) and persulfidation in male reproduction. Sci. Rep..

[22] Pintus E (2023). N-thiocarboxyanhydrides, amino acid-derived enzyme-activated H_2_S donors, enhance sperm mitochondrial activity in presence and absence of oxidative stress. BMC Vet. Res..

[23] Wang J (2018). Hydrogen sulfide as a potential target in preventing spermatogenic failure and testicular dysfunction. Antioxid. Redox Signal..

[24] Zuhra K, Augsburger F, Majtan T, Szabo C (2020). Cystathionine-β-synthase: Molecular regulation and pharmacological inhibition. Biomolecules.

[25] Mao Y-G, Chen X, Zhang Y, Chen G (2020). Hydrogen sulfide therapy: A narrative overview of current research and possible therapeutic implications in future. Med. Gas Res..

[26] Sugiura Y (2005). Cadmium exposure alters metabolomics of sulfur-containing amino acids in rat testes. Antioxid. Redox Signal..

[27] Li G, Xie Z-Z, Chua JM, Wong P, Bian J (2015). Hydrogen sulfide protects testicular germ cells against heat-induced injury. Nitric Oxide.

[28] Gao D-D (2019). Cellular mechanism underlying hydrogen sulfide mediated epithelial K+ secretion in rat epididymis. Front. Physiol..

[29] Kadlec M, Ros-Santaella JL, Pintus E (2020). The roles of no and h2s in sperm biology: Recent advances and new perspectives. Int. J. Mol. Sci..

[30] Guo H (2012). Characterization of hydrogen sulfide and its synthases, cystathionine β-synthase and cystathionine γ-lyase, in human prostatic tissue and cells. Urology.

[31] Rahardjo HE, Ückert S, Kuczyk MA, Hedlund P (2023). Expression and distribution of the transient receptor potential cationic channel ankyrin 1 (TRPA1) in the human seminal vesicles. Health Sci. Rep..

[32] Yetik-Anacak G (2016). Hydrogen sulfide compensates nitric oxide deficiency in murine corpus cavernosum. Pharmacol. Res..

[33] d’Emmanuele di Villa Bianca R (2009). Hydrogen sulfide as a mediator of human corpus cavernosum smooth-muscle relaxation. Proc. Natl. Acad. Sci..

[34] Srilatha B, Adaikan PG, Moore PK (2006). Possible role for the novel gasotransmitter hydrogen sulphide in erectile dysfunction—A pilot study. Eur. J. Pharmacol..

[35] Srilatha B, Adaikan PG, Li L, Moore PK (2007). Hydrogen sulphide: A novel endogenous gasotransmitter facilitates erectile function. J. Sex. Med..

[36] Shukla N (2009). Effect of hydrogen sulphide-donating sildenafil (ACS6) on erectile function and oxidative stress in rabbit isolated corpus cavernosum and in hypertensive rats. BJU Int..

[37] Li J (2011). Endogenous hydrogen sulfide as a mediator of vas deferens smooth muscle relaxation. Fertil. Steril..

[38] Li Y (2012). H2S relaxes vas deferens smooth muscle by modulating the large conductance Ca2+-activated K+ (BKCa) channels via a redox mechanism. J. Sex. Med..

[39] Qi Q (2021). A novel posttranslational modification of histone, H3 S-sulfhydration, is down-regulated in asthenozoospermic sperm. J. Assist. Reprod. Genet..

[40] Xie Z-Z, Liu Y, Bian J-S (2016). Hydrogen sulfide and cellular redox homeostasis. Oxid. Med. Cell. Longev..

[41] Al-Magableh MR, Kemp-Harper BK, Ng HH, Miller AA, Hart JL (2014). Hydrogen sulfide protects endothelial nitric oxide function under conditions of acute oxidative stress in vitro. Naunyn-Schmiedeberg's Arch. Pharmacol..

[42] Predmore BL, Lefer DJ, Gojon G (2012). Hydrogen sulfide in biochemistry and medicine. Antioxid. Redox Signal..

[43] Kimura Y, Goto Y-I, Kimura H (2010). Hydrogen sulfide increases glutathione production and suppresses oxidative stress in mitochondria. Antioxid. Redox Signal..

[44] Mao Z (2019). Pharmacological levels of hydrogen sulfide inhibit oxidative cell injury through regulating the redox state of thioredoxin. Free Radic. Biol. Med..

[45] Chen X (2014). Hydrogen sulfide reduces kidney injury due to urinary-derived sepsis by inhibiting NF-κB expression, decreasing TNF-α levels and increasing IL-10 levels. Exp. Ther. Med..

[46] Zhao Y, Biggs TD, Xian M (2014). Hydrogen sulfide (H_2_S) releasing agents: Chemistry and biological applications. Chem. Commun..

[47] Donald JA (2021). Handbook of Hormones.

[48] Lazado CC, Voldvik V, Timmerhaus G, Andersen Ø (2023). Fast and slow releasing sulphide donors engender distinct transcriptomic alterations in Atlantic salmon hepatocytes. Aquat. Toxicol..

[49] Panthi S, Chung H-J, Jung J, Jeong NY (2016). Physiological importance of hydrogen sulfide: Emerging potent neuroprotector and neuromodulator. Oxid. Med. Cell. Longev..

[50] Zaichko N, Melnik A, Yoltukhivskyy M, Olhovskiy A, Palamarchuk I (2014). Hydrogen sulfide: Metabolism, biological and medical role. Ukr. Biochem. J..

[51] Li K-P, Yang X-S, Wu T (2022). The effect of antioxidants on sperm quality parameters and pregnancy rates for idiopathic male infertility: A network meta-analysis of randomized controlled trials. Front. Endocrinol..

[52] Cilio S (2022). Beneficial effects of antioxidants in male infertility management: A narrative review. Oxygen.

[53] Majumder A (2023). Targeting homocysteine and hydrogen sulfide balance as future therapeutics in cancer treatment. Antioxidants.

[54] Kimura Y, Kimura H (2004). Hydrogen sulfide protects neurons from oxidative stress. FASEB J..

[55] Whiteman M (2004). The novel neuromodulator hydrogen sulfide: An endogenous peroxynitrite ‘scavenger’?. J. Neurochem..

[56] Lu M, Hu L-F, Hu G, Bian J-S (2008). Hydrogen sulfide protects astrocytes against H_2_O_2_-induced neural injury via enhancing glutamate uptake. Free Radic. Biol. Med..

[57] Dutta S (2022). Antioxidant paradox in male infertility: ‘A blind eye’ on inflammation. Antioxidants.

[58] Sadeghi N, Boissonneault G, Tavalaee M, Nasr-Esfahani MH (2023). Oxidative versus reductive stress: A delicate balance for sperm integrity. Syst. Biol. Reprod> Med..

[59] Zhao Y (2016). Hydrogen sulfide and/or ammonia reduces spermatozoa motility through AMPK/AKT related pathways. Sci. Rep..

[60] Szabo C (2014). Regulation of mitochondrial bioenergetic function by hydrogen sulfide. Part I. Biochemical and physiological mechanisms. Br. J. Pharmacol..

[61] Panagaki T, Randi EB, Augsburger F, Szabo C (2019). Overproduction of H_2_S, generated by CBS, inhibits mitochondrial Complex IV and suppresses oxidative phosphorylation in Down syndrome. Proc. Natl. Acad. Sci..

[62] Ning JZ (2018). The protective effects of GYY4137 on ipsilateral testicular injury in experimentally varicocele-induced rats. Exp. Ther. Med..

[63] Gualtieri R (2021). Sperm oxidative stress during in vitro manipulation and its effects on sperm function and embryo development. Antioxidants.

[64] Ismail AA, Abdel-Khalek A, Khalil W, El-Harairy M (2020). Influence of adding green synthesized gold nanoparticles to tris-extender on sperm characteristics of cryopreserved goat semen. J. Anim. Poult. Prod..

[65] Gacem S, Catalán J, Yánez-Ortiz I, Soler C, Miró J (2021). New sperm morphology analysis in equids: Trumorph^®^ vs eosin-nigrosin stain. Vet. Sci..

[66] Bassiri F, Tavalaee M, Shiravi A, Mansouri S, Nasr-Esfahani M (2012). Is there an association between HOST grades and sperm quality?. Hum. Reprod..

[67] Tanhaei Vash N, Nadri P, Karimi A (2022). Synergistic effects of myo-inositol and melatonin on cryopreservation of goat spermatozoa. Reprod. Domest. Anim..

[68] Kiani-Esfahani A, Tavalaee M, Deemeh MR, Hamiditabar M, Nasr-Esfahani MH (2012). DHR123: An alternative probe for assessment of ROS in human spermatozoa. Syst. Biol. Reprod. Med..

[69] Guthrie H, Welch G (2006). Determination of intracellular reactive oxygen species and high mitochondrial membrane potential in Percoll-treated viable boar sperm using fluorescence-activated flow cytometry. J. Anim. Sci..

[70] Aitken RJ, Wingate JK, De Iuliis GN, McLaughlin EA (2007). Analysis of lipid peroxidation in human spermatozoa using BODIPY C11. MHR Basic Sci. Reprod. Med..

[71] Nur Z, Zik B, Ustuner B, Sagirkaya H, Ozguden C (2010). Effects of different cryoprotective agents on ram sperm morphology and DNAintegrity. Theriogenology.

[72] Agdam HR, Razi M, Amniattalab A, Malekinejad H, Molavi M (2017). Co-administration of vitamin E and testosterone attenuates the atrazine-induced toxic effects on sperm quality and testes in rats. Cell J. (Yakhteh).

[73] Sadeghi M (2023). Developmental competence of IVF and SCNT goat embryos is improved by inhibition of canonical WNT signaling. PLoS One.

[74] Habibi R (2018). Functional characterization of NANOG in goat pre-implantation embryonic development. Theriogenology.

